# Medical debt during epidemics: A case for resolving the situation in low- and middle-income countries such as Kenya

**DOI:** 10.12688/wellcomeopenres.18403.1

**Published:** 2022-10-04

**Authors:** Tom Obengo

**Affiliations:** 1 Department of Medicine, University of Cape Town, Cape Town, South Africa; 2 Ethox Centre, University of Oxford, Oxford, UK

**Keywords:** Medical debt; human dignity; epidemic ethics; health care funding.

## Abstract

This paper evaluates the problem of medical debt in Kenya during the COVID-19 pandemic. The medical debt problem is compounded during pandemics such as COVID-19 when patients seek treatment and end up in insurmountable debt because illnesses related to the pandemic are not covered by the Kenyan National Health Insurance Fund (NHIF), the public health coverage body under government control. As a result, discharged patients may be detained in hospitals and dead bodies are locked away in mortuaries, until relatives and friends fundraise and clear the bills. Apart from causing vulnerability, fear, and emotional stress among the poor, this practice leads to a growing lack of trust in the healthcare system, with patients deliberately avoiding hospitals whenever they suspect they have COVID-19. The resulting vicious cycle makes healthcare more inaccessible by limiting the choices that people may have.

User fees, which were introduced in all public health facilities by the Kenyan government as part of a World Bank prescription for cost-sharing, normally affect more women than men. Although Kenya has implemented a general waiver system in public hospitals for those who cannot pay their medical bills, the process of obtaining this waiver can be burdensome, demeaning, and dangerous for the health of the patients. This undermines the government’s commitment to the provision of equitable and affordable health care for the citizens.

In this article, the problem of medical debt in Kenya is addressed as a multi-faceted problem drawing on issues of justice and fairness, human dignity, good governance, the interplay between global and local policies, as well as politics and law. It argues that it is in the best interest of Kenya and other African countries to ensure that public health coverage covers pandemics so that the majority poor can afford and access healthcare.

## Introduction

When the World Health Organisation (WHO) declared COVID-19 a global pandemic in March 2020, it emphasized the importance of ensuring that all patients around the world received care ( WHO, 2020). Yet as the sick in Kenya sought treatment in public hospitals, they quickly learned that the National Hospital Insurance Fund (NHIF), which would normally pay at least part of a hospital bill for its members, did not cover COVID-19-related cases, and that patients would have to pay from their pockets. Having gone to hospital due to a pandemic- related emergency, some patients were detained by hospitals until their relatives would pay up. There were also cases of dead bodies retained in hospital mortuaries, awaiting payment of bills by families, relatives, friends, and other social networks. This led to poor people avoiding COVID-19 tests and hospitals for fear of detention over accruing bills. I argue that this compromised public health responses to the pandemic, including potentially directly causing increased suffering and death. Case studies used in this paper were publicly available cases or written informed consent was provided by the deceased’s family. Ethical approval was not required for this paper since it is a conceptual and review paper.

### The medical debt situation in Kenya/Africa

In Kenya’s healthcare system there are public and private healthcare facilities ( Obengo, 2020). ^1^ The latter is funded by private healthcare insurance and predominantly services the wealthy. All the other persons rely on the public healthcare system which, in turn, relies on a mixture between self-financing and the National Hospital Insurance Fund (NHIF). The NHIF is a Kenya government corporation that was established in 1966 as a department under the Ministry of Health. It was set up by an Act of Parliament to help the government in taking care of funding the health needs of the population. Currently an NHIF Act No 9 of 1998 governs the fund ( Government, 2012 [1998]). The Fund’s core mandate is to provide medical insurance cover to all its members and their declared dependants (spouse and children). The NHIF membership is open to all Kenyans who have attained the age of 18 years, mostly if they are employed, although it is also open to people who are either in small businesses or unemployed. To provide customer service, NHIF has 95 fully autonomous branches and satellite offices across the country ( NHIF, 2022).

The problem of medical debt is not solely an African issue, but one that affects people in countries across the globe, including for instance in the United States of America (USA). The prevalent experience among many is that a patient who becomes critically ill is taken to a healthcare facility by family, relatives, or friends who care more about the patient’s need for medical attention than about the means by which the bills will get covered. As soon as the patient gets admitted for observation, tests, and treatment, the bills begin to accumulate. Some private hospitals deny admission to patients who do not pay deposits, even if the situation is a medical emergency, and send them to seek treatment elsewhere. Although a family may not normally take someone to a private healthcare facility if they do not have insurance, they may do so if it were an emergency, such as a sudden illness during an epidemic. Whether in a public health facility or in a private one, the bills are extremely difficult – sometimes impossible - for the poor to pay. In some cases, even those with medical cover get stranded when they reach a stage of inability to pay for services after they exhaust the limits of their cover.

In the Kenyan context, whenever a patient dies during a course of treatment, leaving behind unpaid bills, the family is asked to pay the bill before the body is released for burial ( Oyugi, 2019). The practice benefits from the assumption that Africans place premium value on providing a culturally befitting burial to their dead and would go to any extent, including selling their only remaining family resource or property, to obtain a dead body for burial. During epidemics when more deaths than usual occur, the practice, if not monitored, may lead to congestion in the mortuaries. It may also lead to social unrest among communities whose relatives remain unburied due to indebtedness to hospitals and complicate the grief and loss experienced by families.

One of the recent public cases of medical debt in Kenya in relation to the COVID-19 pandemic was that of Dr Lincoln Khasakhala ^2^, a medical doctor and a Senior Lecturer at the University of Nairobi ( Mutai, 2022). The university had provided him and his family with medical cover. In March 2021 he contracted COVID-19 while carrying out healthcare duties and infected his wife. They were both hospitalised at the Nairobi Hospital where his wife died soon after admission. After his wife’s burial, Dr Khasakhala’s condition was complicated by his diabetic condition and a pneumonia attack. On 3 ^rd^ July 2021, while he was unconscious, he was admitted in the hospital’s Intensive Care Unit (ICU) where he underwent treatment, which included weekly dialysis, for six months before his death on 8 ^th^ January 2022. The only dependant the couple left behind was an underage child. By that time, after insurance cover had paid up to the limit of the cover, his outstanding bill had accrued to KES 21,272,326. The family paid KES 2,982,458, leaving a balance of KES 18,081,505 (about US $153,500). The hospital decided not to release the body for burial until the rest of the bill was paid in full ( Musau, 2022). Dr Khasakhala had stayed in a vegetative state until his death. The public university at which he had been a lecturer declined to help meet the bill, saying the doctor should have sought approval before getting admitted, ignoring the fact that the patient had been taken to the hospital unconscious, in critical condition, and in need of emergency attention. The matter reached Parliament’s Health Committee which made an inquiry to determine why the hospital would not release the body for burial. The Committee met the NHIF officials, Dr Khasakhala’s family, and the University of Nairobi, the late doctor’s employer, and resolved that the body be immediately released for burial ( Robi, 2022). Parliament committed to consult with the Ministry of Health on how the hospital would be compensated.

In another case, a young lady close to our family had a sickly and alcoholic brother ^3^ who survived on daily casual jobs. He contracted COVID-19 and was hospitalised at a nearby mission hospital and moved to the High Dependency Unit (HDU) in the same hospital. In order to ensure the patient could benefit for the NHIF cover, and after advice from a hospital administrator, his sister paid all outstanding NHIF fees that the brother had not paid. Two weeks later he died, and his sister promptly informed us, so we went to the hospital to assist her in clearing. The NHIF officials informed us that NHIF does not cover any cases of COVID-19 patients, and the hospital administrators said that the KES 267,000 (about US $2,246) bill had to be paid by the deceased’s relatives and friends before the body would be released for burial. We mobilised relatives, the church, and the community, paid up the bill, and collected the body for burial at a local cemetery. We later learnt from others that NHIF had disappointed them too with regard to COVID-19 patients. NHIF officials also later clarified that hospital admissions based on COVID-19 cases are not covered at all. 

## A review of health care financing in Kenya

There are six ways by which Kenyans take care of their medical bills. The first one is by simply doing nothing. The response of doing absolutely nothing in attempting to pay for medical treatment in the most common among the poor. This is not merely a response born of negligence or irresponsibility, but one of desperation that strips the person of any human dignity left. The person has no money, no property left to sell to meet the medical needs, and no source of income at all. In extreme cases, some such patients would prefer death to life in debt, and even appreciate hospital detention, since there is at least some food provided, instead of being released to go home where nothing is available. The second way is by individuals simply paying from their pocket, which works well for outpatient services and short admissions. The third way is through leveraging social networks which include relatives, workplace colleagues, and friends. This approach works well for those who have some resources of their own and have previously participated in helping out in their social networks. The fourth way is the engagement of the National Hospital Insurance Fund (NHIF) or the newly developed (but not yet implemented) Universal Health Coverage (UHC). Both are state-managed enterprises to which members are invited to pay monthly instalments to purchase of medical cover. The fifth is employer compensation, an arrangement in which the employer commits to pay a specific amount of money towards treatment whenever an employee falls sick or gets hospitalised. This works well when the amounts involved are small and only a few employees fall sick. Employers like it because it enables them to avoid having to pay for regular health insurance for employees. The sixth is the commercial medical insurance cover purchased from established insurance companies in and outside Kenya. This is most preferred by the working middle class and the rich because it is convenient, it gives them preferential treatment, and they can afford it.

## Causes of medical debt in Kenya

### Lack of medical insurance

The majority of Kenyans are not covered by any health insurance policy. Currently, the publicly funded NHIF covers only 15.8% of the country’s population. Only another 4.2% of the Kenyan population have any form of health insurance ( Owino, 2021).

“Health insurance coverage in Kenya remains low and is characterised by significant inequality. In a context where over 80% of the population is in the informal sector, and close to 50% live below the national poverty line, achieving high and equitable coverage levels with contributory and voluntary health insurance mechanism is problematic” ( Kazungu & Barasa, 2017, p. 1175).

In 2015 the Kenyan government expanded NHIF’s benefits from inpatient treatment only, to a coverage that now includes outpatient services. It also introduced a health insurance subsidy programme whereby poor households are identified and given a 100% subsidy on NHIF membership ( Barasa *et al.,* 2018). Both new policies have, however, remained elusive, since they are not implemented in practice, and the subsidy programme has remained inaccessible, since the procedures to enrol are complex and difficult for the poor to manage.

 These challenges associated with lack of access to funded health care led to frustrations during the COVID-19 outbreak when patients with COVID-19 symptoms attended health facilities, expecting that their NHIF cover would take care of bills, only to be disappointed with instructions to pay the costs of treatment from their own pockets. Three important points worth noting here are that a) it would have been reasonable to expect the NHIF to pay for COVID-19 treatment in cases where the presenting patients had paid for general cover; b) that COVID-19-related costs were excluded from NHIF was not communicated clearly to the public ^4^; and c) that the political decision to exclude these costs was strategic and not taken with the interests of the patients or the Kenyan population in mind. The cost of COVID-19 testing in Kenya has not yet been included in the NHIF with only a few people living in hotspot areas receiving free COVID-19 testing. The rest have to pay an estimated cost of US $100 (KES 10,825.00) to get the tests done. This is a difficult undertaking in a society where more than 40% of the population survives on less than US $2 (KES 216.31) a day ( Ouma *et al.,* 2020). 

### Insufficient medical cover levels

Medical cover normally has limits on the benefits that policy holders receive. Upper limits are set with the understanding that a patient’s treatment costs will generally fall within the set limit. Yet no one can accurately predict for sure what kind of disease is likely to attack them, and how much the treatment is likely to cost. To the extent to which medical bills grow beyond the health insurance cover limits, the medical cover is insufficient. If a sudden illness takes a patient to a hospital for a possible emergency admission, treatment costs can quickly exhaust the cover within a few days. By the second week the family is dipping into their savings which may also get exhausted by the third or fourth week. If the patient stays hospitalised for a month or two, the family’s resources may be completely drained, and they have to appeal for help from relatives, friends, and workplace colleagues. These networks may help significantly if the patient has also been of help to others in similar situations in the past, which is only possible if they are employed or in a profitable business. This means the poor rarely benefit significantly from social networks when they find themselves sick and hospitalised. The onset of an epidemic such as COVID-19 only aggravates such experiences.

Understandably, medical cover has to have an upper limit beyond which no further payments may be made. During epidemics such as COVID-19, medical bills become extremely challenging and aggravate poverty. In situations of inequality where medical debt literally steals food out of the mouths of entire families, or where the adequate care of patients is clearly in the public interest, more work needs to go into determining to what extent medical debt is appropriate or acceptable. Furthermore, in situations where the creation of medical debt clearly leads to unethical practices - such as keeping patients or human bodies hostage until the debt is settled - there similarly are questions about the role of government in protecting citizens during medical emergencies. As discussed below, I will argue that the government and the private sector should come up with a new approach.

### Poverty and unemployment

In Kenyan public discourse, the problem of medical debt is sometimes considered a personal problem, and one that is a consequence of people’s own responsibility ( Goff, 2021). However, being and staying poor should not result in the poor being blamed for being irresponsible. Rather poverty has more to do with structural conditions and the burdens placed on the poor through bad governance. Although the causes of poverty are structural and multifaceted, bad governance is a leading cause because it influences and controls how citizens access and enjoy resources. In other words, it is wrong to consider the problem of medical debt as being a problem that affects the poor only, or that is the responsibility of those individuals that are unable to pay their bills. The problem of medical debt in Kenya is partly a direct consequence of global health inequality as well as particular political decisions and behaviours in Kenya that keep poor people poor. It would be important for the Kenya government to minimise out of pocket payments for health care which disproportionately affect the poor who can least afford it ( Office, 2014). Where this has not been taken care of, especially in LMICs, the costs have ended up as informal charges at the local level ( Chapman *et al.,* 2018).

Furthermore, medical debt has consequences not just for the patients and their extended family who need to cover the costs, but also for the wider community and the public. In particular, in the context of the COVID-19 pandemic, there was a global emphasis on containing the spread of infection ( WHO, 2020). However, as news spread in Kenya that COVID-19-related healthcare costs would not be covered by the NHIF, people started avoiding tests and hospitals. In such contexts medical debt can be considered the mortal enemy of the patient, the physician, the hospital, the community, the state, and the nation ( Goff, 2021). In pandemics, governments have a responsibility to develop policies that focus on saving the lives and well-being of the worst off in the country. Inequalities exacerbated by the pandemic need to be addressed in order for the country to achieve universal health care. As has been suggested, to be consistent with a human rights approach, access to essential health services and public health protections should be based on a true universality covering all residents of a country regardless of their legal status. Furthermore, it should be a legal entitlement over which a citizen may seek redress to challenge failure to provide the statutory benefits ( Chapman *et al.,* 2018). These measures could include the use of emergency funds to pay off COVID-related treatments in public hospitals where the poor seek health care.

## How medical debt compromises health care during epidemics

There are four main ways by which medical debt compromises health care during epidemics such as COVID-19. The first one is that medical debt causes patient fear and avoidance of hospitalisation even when critically ill. Even when a person suspects they may be infected by COVID-19, they may also know that the treatment is likely to be expensive and may result into their detention if not paid in full. This leads to people avoiding tests and treatment and such a patient will do what they can to avoid getting hospitalised. In situations where many such treatment-avoiding patients exist, more people will get infected, and the disease will continue to spread. In this sense, medical debt is a serious enemy of public health in the country – and potentially global public health as well.

The second way by which medical debt compromises health care during epidemics is that it increases the numbers of indebted hospitals that owe large sums of money to organisations that supply medicines and equipment, and sometimes even banks that pay salaries. Hospitals are high-expenditure facilities which will, sometimes, receive supplies on credit, with the assumption that patient fees will be used to service the debts. When laboratory tests and medical services are not paid for, the hospital has to remain in debt to its creditors, just like the patient is to the hospital. Where several hospitals are indebted, public emergencies become difficult to manage, and more deaths occur than would have been if hospitals could meet their debts.

The third way is through an emergence of mistrust between patients and health care providers. When public health experts inform the people that an epidemic has set in, people treat the information with suspicion. Recipients of public information perceive health care professionals as people whose help will not count where it matters most. The public assumes that costs of treatment will be harder for them to meet during an epidemic, hence the reluctance to follow the expert instructions from health care professionals. In other words, health care services will be for those who can afford and will be a risk for those who cannot afford to pay; a risk in relation to inability to pay, potentially leading to hospital detention. Conversely, patients will not be trusted to share accurate information with others, since one would not want to be perceived to have misadvised a neighbour or a relative into seeking medical care that led to detention in a hospital. Such mistrust leads to slowness in dealing with a pandemic because people will not seek care.

The fourth way is in the non-compliance with public health requirements by the general public. This comes about as a follow-up from the mistrust between caregivers and patients. People who cannot afford to pay out-of-pocket perceive the health care system as non-beneficial to them, and whatever instructions they are given about managing their health in an epidemic are given for the benefit of other people, not themselves. For example, during the COVID-19 pandemic, the Kenyan public was instructed that, when feeling certain persistent symptoms, they should go to hospital for tests and treatment. But patients living in poverty and in fear of medical debt are unlikely to comply and, instead, seek alternative remedies which may not necessarily be effective. Such non-compliance sometimes facilitates the spread and gravity of the pandemic.

## Detention of discharged patients in hospitals due to medical debt

It has become common practice in Kenya that discharged patients who cannot pay up their treatment bills get detained in the hospitals until the bills gets paid, one way or another, in full. During such detention the family and relatives of a discharged patient are informed that they must pay the entire bill before the patient is released. Detained patients occupy space that the hospital could use in serving other patients. There are dangers that come with this practice as patients become desperate for freedom. For example, in 2017 a detained patient at Kenyatta National Hospital committed suicide when he could not pay his treatment bills ( Oyugi, 2019). Furthermore, the hospital executive and staff do not have a legal right to detain people; if there is a problem of unpaid debts, then the police and the courts of law should be involved. By allowing hospitals to take on the functions of the police and the courts of law, we create lawlessness in the sense of every indebted institution taking the law into their own hands. Finally, when patients are kept hostage in the hospital, they no longer receive care from hospital staff (they are not patients after all), nor are they entitled to a bed, or any other form of support, although they receive minimal food rations. In addition to suffering from neglect, patients may also acquire new and deadly infections, such as COVID-19, during their detention in the hospital.

In order to release patients or bodies from hospitals, family members have an option of raising funds from social networks. Poor people in Africa in general, and in Kenya in particular, have developed extended networks through which they support each other in times of dire need. Since the onset of COVID-19, for example, small-scale businesses in the slums of Nairobi had fewer customers and less earnings. Those who were members of small savings groups received some relief during hard times, including sicknesses. Such groups meet regularly, either weekly or monthly, for sessions of what is known as ‘table banking’ during which “…each member contributes an agreed amount of money, which can then be loaned out within the group” ( Njagi, 2021). A member of one such small savings group said, “…the people will not wait for someone to come from outside their communities to solve their problems because they will use the experience that happens within the community to stand for themselves” ( Njagi, 2021), and this applied during COVID-19 illnesses among members whenever medical bills accrued. But, notably, these are people who are running small-scale businesses, profits of which enable them to be credit worthy. In other words, the wealthier one is, the more effectively one can lobby one’s network for funds. But if one is poor one has not ‘reciprocity credit’ to enable such one to benefit from such social groups. This means that poor people in unequal African societies, even at the lowest economic levels, are less likely to be successful.

Ironically, the Constitution of Kenya (2010) guarantees every citizen the highest attainable standard of health which includes the right to healthcare services and prescribes that a person shall not be denied emergency medical treatment. Furthermore, it provides for the freedom and security of every person, specifically stating that no one should be detained without just cause. In the same constitution, every person is guaranteed a right to dignity and the right to freedom of movement. There is no law that provides that failure to pay hospital bills should result in detention. The practice, which is prevalent in both public and private hospitals, is unlawful and disproportionally affects the rights and interests of Kenya’s poorest citizens. During the lockdowns instituted in the face of the COVID-19 pandemic, the poor especially experienced serious socio-economic burdens arising from costs accrued from emergency treatment, especially since no relief was provided despite support from global partners. “For the fight against COVID-19, the Global Fund has awarded US $139 million to Kenya through the COVID-19 Response Mechanism (C19RM) to support the procurement of diagnostics, personal protective equipment and oxygen, and reinforce key investments in health and community systems” ( Director, 11 March 2022). There had been grants from the International Monetary Fund (IMF) as well ( Owino, 1st January 2022). However, these and other funds designated for COVID-19 pandemic could not be accounted for and the Auditor-General reported it as part of a corruption network ( Oketch & Ngugi, 2022).

## Ethical issues arising on medical debt

The problem of medical debt, especially during epidemics, raises critical ethical issues that need the attention of policy makers and leaders. The most prominent of these are issues of justice and fairness. Although these two ideas may seem similar, they are conceptually different. While fairness highlights the quality of showing impartiality with no bias towards anyone, justice focuses on the quality of giving everyone what is due to them. ^5^ In expressing to others how they deserve to be treated, we speak in terms of both justice and fairness. As John Rawls would put it, “…each person is to have an equal right to the most extensive basic liberty compatible with a similar liberty for others” ( Duignan, 2022). Rawls explains, “Social and economic inequalities are to be arranged so that they are both ( *a*) to the greatest benefit of the least advantaged and ( *b*) attached to offices and positions open to all under conditions of fair equality of opportunity” ( Duignan, 2022); ( Rawls, 2002, p. 276). Access to health care in Kenya is not a similar experience for everyone. Indeed, the poor and unemployed majority of the population do not have the same level of liberty as the rich minority do. Medical debt aggravates health inequality. It is a problem that communities have to mobilise their meagre resources for, while also holding the government accountable to resolving. Since governments should promote justice, they should aspire for and work to establish a just society in which “…those who are worst off are prosperous enough to be economically independent” ( Duignan, 2022). In the context of medical debt, especially during epidemics, the worst off, those who are living in extreme poverty and are unemployed, need to benefit from deliberate government affirmative action.

A second ethical issue to consider is human dignity which refers to the idea of recognising and promoting the self-esteem of the human being. ^6^ The import of this in the discussion of medical debt is that, since experiences of disease can be inherently dehumanising, medical debt further aggravates the experience of those who get detained in hospitals after medical discharge, those who cannot bury their dead family members because bodies cannot be released, and those who have to lose even the smallest property to pay for treatment. During an epidemic, any subjection of the poor and unemployed to undignified treatment through medical debt only serves to further ground suspicions about an uncaring health care system and uncaring national government. Any policies drafted as attempts at addressing health care financing should, as a priority, place high premium on the human dignity of the citizens.

The third issue is that of poverty and vulnerability, which affects the majority of Kenyans who cannot afford medical cover. Medical debt is a poverty-related problem that exacerbates vulnerabilities when a pandemic strikes. No government should be comfortable when the majority of its citizens have no ease of access to health care, and when some have to get detained in hospitals because of unpaid bills. In the same way foreign aggression might cause vulnerability among homes on contested borders of countries and ignites attention from the central government, medical debt causes vulnerability among the worst off and should attract the attention of the health administrators and policy makers. Leaving anyone vulnerable exposes everyone and makes it more difficult to fight a pandemic since the rest of the population becomes vulnerable too.

A fourth issue is the emergence of a discriminatory health care system in which a few enjoy the best health care because they can either pay out of pocket or purchase reliable medical cover, while the rest struggle to afford and access the same. A discriminatory system is morally bad because it segregates between those who have and those who do not have, those who can access treatment and those who cannot access it, those who will live and those who should die. Such a system recognises a category of citizens as worthier than others of medical services. During epidemics, a discriminatory system creates desperation and resignation in some while giving hope to others. In Kenya, while insurance companies sent their subscribers to the best hospitals for COVID-19 tests, and while those who tested COVID-positive had their treatment covered, the worst off such as those with medical debts simply stayed away and let disease take its course. Discriminatory practices, whether based on class, race, ethnicity, or gender, almost always hurt the most vulnerable members who, ironically, are in more need of government protection. In this case, discriminatory practices in the health care system leads to the inaccessibility of health care by the poor.

## Politics, law, and governance

While analysing the problem of medical debt in Kenya, I argue that the *status quo* is unfair, unjust, and illegal, resulting in a proverbial time-bomb which may result in significant civil unrest. First, I have pointed out the challenge of bad policies such as the one in which Kenya’s NHIF covers some medical procedures and types of treatment but leaves out others such as COVID-19. It is difficult to understand why a government-sponsored cover would decline to cover COVID-19 treatment in any hospital in the country. Bad policies such as the kind that govern NHIF make it difficult to alleviate the dire situations in which the poor find themselves and need to be reviewed in favour of the poor and the unemployed.

The second challenge arises from the broader issue of government corruption. When COVID-19 broke out a number of weaknesses in Kenya’s health care system were exposed. One example is the shortage resources; when COVID-19 hit, the country had only 256 ventilators and 537 ICU beds in a population of 53 million ( Mohiddin & Temmerman, 2020). Funds were made available to increase the Kenyan healthcare system’s ability to deal with the pandemic, but rather than strengthening the healthcare system, entrenched corruption and mismanagement led to the creation of a new group of “COVID millionaires” ( Oketch & Ngugi, 2022). According to some accounts, KES 17 billion (about US $144,435,000) worth of stocks at the Kenya Medical Suppliers Agency (KEMSA) were unaccounted for ( Oketch & Ngugi, 2022). Earlier in January, Parliament had begun investigating a scandal involving the loss of KES 7.8 billion (about US $66,270,180) at the same agency. The funds had been designated for purchasing personal protective equipment when the epidemic broke out in 2020, but these were never supplied, unnecessarily exposing Kenyan healthcare workers to COVID-19. The scandal involved politicians and their allies ( Oketch & Ngugi, 2022). Currently, NHIF is in another scandal after it paid KES336.3 million (about US $2,857,300) to a law firm that had not been pre-qualified to bid for tenders with health providers ( Oketch & Mwere, 2022). Such high levels of official corruption destroy trust among the global partners in the health sector and limit capacities to develop interventions to help with medical debt.

Closely related to corruption is a serious lack of transparency and accountability in the use of funds designated for health care and in government policy development. A case in point is the stealth with which the COVID-19 exemption from the NHIF was published. In addition to addressing corruption and ensuring there is accountability in the use of funds, there must also be transparency about what such funds will and will not cover in relation to COVID-19. Patients and their relatives should not be learning about what will or will not be covered by the NHIF during treatment or, worse, after discharge. Political processes are kept from the public eye until a scandal breaks out. In this case, information about the limits on NHIF cover for COVID-19 was not available to the public until scandals broke out. Such conditions compromise the government’s ability to intervene in cases of excessive medical debt.

### Limitations of this study

Since the study was conceptual, it could not bring out empirical data on the current medical debt crisis among patients and their families in Kenya.

### Potential bias

A critical approach from a moral philosopher may ignore the strengths of the existing health financing system.

## Conclusions

The COVID pandemic has highlighted and exacerbated Kenya’s deep-seated health inequalities. To address such inequalities, it has been suggested that Kenya should consider a universal, tax-funded mechanism that ensures revenues are equitably and efficiently collected, and everyone, including the poor and those in the informal sector, is covered ( Kazungu & Barasa, 2017, p. 1175). This requires prudent collection and management of taxes. In order to stop the detention of discharged patients and the locking up of dead bodies, Kenya needs to strengthen re-insurance policy and practice so that hospitals can recover debts that cannot be paid by patients.

Lessons from the COVID-19 pandemic should encourage the government to pull resources even if it means cutting entertainment budget and other less essential expenditures in government to expand the NHIF scheme to cover more people, more services, and treatments (which would include but not limited to COVID-19 testing and management). This would consequently result in the aversion of a looming crisis in the health care system for not only COVID-19 pandemic but also future epidemics that might affect the country.

Of course, related regulations and guidelines will have to be developed and implemented carefully to avoid fraudulent avoidance of bills. Parliament also needs to legislate new and elaborate laws to strengthen Kenya’s progressive constitutional provisions regarding health care. Researchers need to investigate the experiences of people who have been detained in hospital due to unpaid bills. Such research will inform additional policy development with regard to matters of psychological support for poor patients, the strengthening of administrative systems in hospitals to include social work. Lastly, the problem of medical debt needs to be tabled for discussion in government meetings, in parliament, in insurance company meetings, and other corporate establishments that engage in social corporate responsibility. This is critical not just to promote health equality *per se*, but also to mitigate the effect of future disease outbreaks and epidemics.

## Data Availability

No data are associated with this article.

## Footnotes

^1^ Kenya’s healthcare system and classification of facilities is a subject I have covered in fair detail on page 33 of my doctoral dissertation titled *A Utilitarian Assessment of the Relevance of Genetic Therapies for HIV-AIDS in Africa, with Special Reference to the Situation in Kenya* which I completed in the Department of Philosophy at the University of Stellenbosch ( Obengo, 2020).

^2^ This case was widely reported in the mainstream media in Kenya in January 2022 and caused public outcry. The body was released for burial after the Kenya Medical Practitioners and Dentists Board and the courts of law ruled for the release ( Mutai, 2022).

^3^ The lady, whose identity remains confidential, gave both verbal and written permission to use the story of her experience with managing her deceased brother’s sickness and death as part of this paper.

^4^ In order to ascertain the exclusion of COVID-19 from NHIF cover I initiated an email communication between the Customer Care Manager at NHIF office ( customercare@nhif.or.ke) and I ( obengotdrh@gmail.com) on 1 ^st^ September 2022 in which I asked the official, “Does NHIF provide cover for a patient admitted in a hospital due to COVID-19-related illness?” and she replied, “It is not covered by NHIF.”

^5^ These are important, and very brief conceptions of some very large and complex concepts, and I am only briefly highlighting their areas of focus in the broadest sense.

^6^ This is an important concept which I am only briefly highlighting in a broad sense.
