# Supplementary material for: Medical debt during epidemics: A case for resolving the situation in low- and middle-income countries such as Kenya
Source: Wellcome Open Res. 2023 Jul 27;7:245. Originally published 2022 Oct 4. [Version 2] doi: 10.12688/wellcomeopenres.18403.2 (PMC10616656; doi:10.12688/wellcomeopenres.18403.2)
Supplement: Peer review history

# Reviewer report: Reviewer response for version 2

**DOI:** 10.21956/wellcomeopenres.21914.r68291

**Published:** 2023-10-30

**Authors:** Jasper Ubels

**Affiliations:** 1 German Cancer Research Center, Heidelberg, Germany

The paper seems to hold the middle between a narrative review and a policy paper. Through interesting examples and in-depth knowledge about the Kenyan health care system, the author provides a powerful message in how the system is currently unjust and has a large socioeconomic impact, particularly on the most vulnerable in society. This also has the consequence that this paper is not a classic research article, since it does not have a method section and it cannot be replicated.

 The paper is an interesting read and I only have minor comments.

 My first minor comment is that there seems to be some duplication of arguments within the paper. For example, the issue of corruption is mentioned at the end of the section “Detention of discharged patients in hospitals due to medical debt” and in the section “Politics, law, and governance”. Similarly, throughout the article, multiple times it is mentioned that people avoid the healthcare system due to medical debt and its consequences (e.g. first paragraph in section “How medial debt compromises health care during epidemics”, the fourth issue in the section “Ethical issues arising on medical debt”, the last paragraph in the section “Poverty and unemployment”). Another example is the link between poverty and medical debt, which is discussed in the third issue in the section “Ethical issues arising on medical debt” and the section “Poverty and unemployment”.

 These are a couple of examples. I think that the argument in this article could be made more concise by carefully checking such duplications and potentially reorganize parts of the paper.

 My second minor comment is related to the title of the section “A review of health care financing in Kenya”. This section might be misunderstood by some readers as a literature review. To prevent any misunderstanding, it might be a good idea to rename this section (e.g. Managing medical bills).

Is the work clearly and accurately presented and does it cite the current literature?

Yes

If applicable, is the statistical analysis and its interpretation appropriate?

Not applicable

Are all the source data underlying the results available to ensure full reproducibility?

No source data required

Is the study design appropriate and is the work technically sound?

Yes

Are the conclusions drawn adequately supported by the results?

Yes

Are sufficient details of methods and analysis provided to allow replication by others?

No

Reviewer Expertise:

Health economics, patient-reported outcomes, capability approach.

I confirm that I have read this submission and believe that I have an appropriate level of expertise to confirm that it is of an acceptable scientific standard.

# Reviewer report: Reviewer response for version 1

**DOI:** 10.21956/wellcomeopenres.20403.r56831

**Published:** 2023-06-01

**Authors:** Gianpaolo Tomaselli

**Affiliations:** 1 University of Malta, Msida, Malta

This conceptual paper provides an interesting overview of the problems related to medical debt in Kenya during the Covid-19 pandemic. However, there are certain elements that need to be improved in order to consider this work as publishable:

1. Lack of references. Some sections (e.g. A review of health care financing in Kenya - just to mention one) do not include any references at all. I would suggest the authors to go through the whole paper and include adequate references where needed.
2. I would suggest the authors to include tables summarizing main points. E.g. for the health care financing section, or for the causes of medical debt. Tables/figures would certainly help to make the content clearer and more organized. 
3. English language. The language used needs to be more professional and technical. Please avoid simplifications such as "The first one is by simply doing nothing." Please go through the paper and revise English language where needed. 

Is the work clearly and accurately presented and does it cite the current literature?

Partly

If applicable, is the statistical analysis and its interpretation appropriate?

Not applicable

Are all the source data underlying the results available to ensure full reproducibility?

No source data required

Is the study design appropriate and is the work technically sound?

Partly

Are the conclusions drawn adequately supported by the results?

Partly

Are sufficient details of methods and analysis provided to allow replication by others?

No

Reviewer Expertise:

Health Systems Management & Leadership, Health Policy, Corporate Social Responsibility, Person-centered Care, Ethics.

I confirm that I have read this submission and believe that I have an appropriate level of expertise to confirm that it is of an acceptable scientific standard, however I have significant reservations, as outlined above.

# Response

**Published:** 2023-07-22

**Authors:** Tom J. Obengo

**Affiliations:** Department of Medicine, University of Cape Town, Cape Town, Western Cape, South Africa

Thank you for this helpful review. In particular, the suggestion for additional referencing has been taken seriously and applied in selected areas of the article.

# Reviewer report: Reviewer response for version 1

**DOI:** 10.21956/wellcomeopenres.20403.r57197

**Published:** 2023-06-01

**Authors:** Alyssa Parpia

**Affiliations:** 1 Institute of Health Policy, Management, and Evaluation, University of Toronto, Toronto, Ontario, Canada

This article outlines the extent of medical debt in Kenya and how the issue has been exacerbated by the ongoing COVID-19 pandemic. The article is not sufficiently referenced and I suggest reviewing the literature on medical debt and epidemics in general (not necessarily specific to COVID-19 or Kenya) for papers to add to the discussion.

 An example of a paper that should be referenced:

 Roberton, T., Carter, E. D., Chou, V. B., Stegmuller, A. R., Jackson, B. D., Tam, Y., ... & Walker, N. (2020). Early estimates of the indirect effects of the COVID-19 pandemic on maternal and child mortality in low-income and middle-income countries: a modelling study. *The Lancet Global Health*, *8*(7), e901-e908.

 In particular, the section on how medical debt compromises health care during epidemics should include references for the way in which issues associated with medical debt have been magnified by epidemics in lower income countries.

 This work is at the intersection of research and advocacy and effectively argues the case that the medical debt situation in Kenya is entirely unsustainable and predatory.

 The noted limitation of not providing any empirical data should be expanded upon as it is a major limitation of the paper. My main concern with this manuscript is the limited evidence provided to back up statements. Additionally, potential ideas for alleviating medical debt should be put forth.

Is the work clearly and accurately presented and does it cite the current literature?

Partly

If applicable, is the statistical analysis and its interpretation appropriate?

Not applicable

Are all the source data underlying the results available to ensure full reproducibility?

Partly

Is the study design appropriate and is the work technically sound?

Partly

Are the conclusions drawn adequately supported by the results?

Partly

Are sufficient details of methods and analysis provided to allow replication by others?

Yes

Reviewer Expertise:

Epidemiology, Health Policy, Public Health

I confirm that I have read this submission and believe that I have an appropriate level of expertise to confirm that it is of an acceptable scientific standard, however I have significant reservations, as outlined above.

## References

[1] : Early estimates of the indirect effects of the COVID-19 pandemic on maternal and child mortality in low-income and middle-income countries: a modelling study. *The Lancet Global Health* .2020;8(7) : 10.1016/S2214-109X(20)30229-1 e901-e908 10.1016/S2214-109X(20)30229-1 PMC721764532405459

# Reviewer report: Reviewer response for version 1

**DOI:** 10.21956/wellcomeopenres.20403.r56829

**Published:** 2023-06-01

**Authors:** Lauren Paremoer

**Affiliations:** 1 Political Studies Department, University of Cape Town, Rondebosch, Western Cape, South Africa

The paper makes an important contribution to debates about the feasibility of assuming that implementation of universal health coverage schemes will necessarily translate into improved access to good quality health care services or better health outcomes. It suggests, through the use of some very powerful examples, that exactly the opposite can happen: UHC schemes may introduce their own barriers to care. I think this paper should be indexed but would suggest that the argument could be strengthened by doing the following:

- The article draws on lived experiences and conversations/interviews. However, no quotes from these interactions are included in the text as a way of substantiating some of the claims put forward.
- The distinction between universal health coverage and universal health care is an important one, and the author should include some discussion of this. For example, some of the critiques of universal health coverage in this paper (e.g. that the coverage model contributes to the commercialisation and financialisation of health care) could be substantiated by referencing similar critiques that have been put forward by proponents of universal health care.
- The introductory sections of this paper could benefit from a brief description of the historical trajectory of health care commercialisation and privatisation in Kenya. This would allow the reader to have some sense of the extent to which some of the practices for managing medical debt during Covid-19 that are mentioned in the paper may have their antecedents in older practices.
- The use of Rawls could be explained a little more; the author should describe why they think this is a compelling theoretical framework to use for explaining the dynamics of this specific case study. Given that the study is a self-describe “conceptual study” and based on the contributions of a specific philosopher (Rawls) the author could flag this more definitively earlier on in the article.
- Towards the end of the paper the author makes a very important point about the need to develop systems/procedures for making decisions about the basic forms of care - both old and new - that UHC programmes should be compelled to cover during health emergencies and pandemics. Given the likelihood of future pandemics/PHEICs, this challenge must be addressed by creating fair, just, transparent, and accountable decision-making procedures. It would be interesting if the author could comment on the problems Kenya experienced in this respect during Covid-19 and what reforms, if any, this has prompted in implementing/redesigning UHC.

Is the work clearly and accurately presented and does it cite the current literature?

Partly

If applicable, is the statistical analysis and its interpretation appropriate?

Not applicable

Are all the source data underlying the results available to ensure full reproducibility?

Partly

Is the study design appropriate and is the work technically sound?

Yes

Are the conclusions drawn adequately supported by the results?

Partly

Are sufficient details of methods and analysis provided to allow replication by others?

Partly

Reviewer Expertise:

Global Health Governance, UHC, social movements

I confirm that I have read this submission and believe that I have an appropriate level of expertise to confirm that it is of an acceptable scientific standard, however I have significant reservations, as outlined above.

# Response

**Published:** 2023-07-22

**Authors:** Tom J. Obengo

**Affiliations:** Department of Medicine, University of Cape Town, Cape Town, Western Cape, South Africa

Thank you for this helpful review.

# Reviewer report: Reviewer response for version 1

**DOI:** 10.21956/wellcomeopenres.20403.r53961

**Published:** 2023-02-17

**Authors:** Zhi Xiang Ng

**Affiliations:** 1 School of Biosciences, Faculty of Science and Engineering, University of Nottingham Malaysia, Semenyih, Malaysia

This article has addressed the importance of considering socioeconomic determinants and consequences of the COVID-19 pandemic in LMIC, especially for rural, vulnerable, and/or marginalised populations. Nevertheless, author is advised to refer the following recently published article discussing the priorities in solving the COVID pandemic problem in low and middle-income countries (LMIC). As the current manuscript focused on Kenya population, it would provide further justification to one of the pressing post-pandemic problem like medical debt.

*“Polašek O, Keri Wazny K, Adeloye A, Song P, Chan KY, Bojude DA, Ali S, Bastien S, Becerra-Posada F, Borrescio-Higa F, Sohaila Cheema12, Cipta DA, Cvjetković S, Castro LD, Ebenso B, Femi-Ajao O, Ganesan B, Glasnović A, He L, Heraud JM, Igwesi-Chidobe C, Iversen PO, Jadoon B, Karim AJ, Khan J, Biswas RK, Lanza G, Lee SWH, Li Y, Liang LL, Lowe M, Islam MM, Marušić A, Mshelia S, Manyara AM, Htay MNN, Parisi M, Peprah P, Sacks E, Akinyemi KO, Shahraki-Sanavi F, Sharov K, Rotarou ES, Stankov S, Supriyatiningsih W, Chan BTY, Tremblay M, Tsimpida D, Sandro Vento S, Glasnović JV, Wang L, Wang X, Ng ZX, Zhang J, Zhang Y, Campbell H, ChopraM, Cousens S, Krstić G, Macdonald C, Mansoori P, Patel S, Sheikh A, Tomlinson M, Tsai AC, Yoshida S, Rudan I; on behalf of the International Society of Global Health (ISoGH). Research priorities to reduce the impact of COVID-19 in low- and middle-income countries. J Glob Health 2022;12:09003.”*

 Major issue:

 More detailed elaboration on the limitation of current study and potential bias section. It would be more informative to provide more in depth discussion on the importance of empirical data when compared to conceptual discussion and how a critical approach from a moral philosopher may ignore the strengths of the existing health financing system.

 Minor issue:

 When writing a scientific article, author should avoid using first party pronunciation like “I”, “we”, “you”, etc.. Author is advised to check the whole manuscript and revise those first party to third party pronunciation.

Is the work clearly and accurately presented and does it cite the current literature?

Partly

If applicable, is the statistical analysis and its interpretation appropriate?

I cannot comment. A qualified statistician is required.

Are all the source data underlying the results available to ensure full reproducibility?

Partly

Is the study design appropriate and is the work technically sound?

Partly

Are the conclusions drawn adequately supported by the results?

Partly

Are sufficient details of methods and analysis provided to allow replication by others?

Partly

Reviewer Expertise:

Epidemiology

I confirm that I have read this submission and believe that I have an appropriate level of expertise to confirm that it is of an acceptable scientific standard, however I have significant reservations, as outlined above.

## References

[1] : Research priorities to reduce the impact of COVID-19 in low- and middle-income countries. *J Glob Health* .2022;12: 10.7189/jogh.12.09003 09003 10.7189/jogh.12.09003 35475006PMC9010705

# Response

**Published:** 2023-07-03

**Authors:** Tom J. Obengo

**Affiliations:** Department of Medicine, University of Cape Town, Cape Town, Western Cape, South Africa

I thank the reviewer for suggesting a more detailed elaboration on the limitation of current study and potential bias section. I will use the suggestion to revise the relevant section.  I will also look into the use of pronouns and revise as may be found necessary. My use of first person pronouns was a result of recommendations from other senior scholars.
